# Multifunctional Nanodrug Mediates Synergistic Photodynamic Therapy and MDSCs‐Targeting Immunotherapy of Colon Cancer

**DOI:** 10.1002/advs.202100712

**Published:** 2021-05-21

**Authors:** Dongbing Ding, Huihai Zhong, Rongpu Liang, Tianyun Lan, Xudong Zhu, Shengxin Huang, Yong Wang, Jun Shao, Xintao Shuai, Bo Wei

**Affiliations:** ^1^ Department of Gastrointestinal Surgery The Third Affiliated Hospital of Sun Yat‐sen University Guangzhou 510630 China; ^2^ PCFM Lab of Ministry of Education School of Materials Science and Engineering Sun Yat‐sen University Guangzhou 510275 China; ^3^ Central Laboratory The Third Affiliated Hospital of Sun Yat‐sen University Guangzhou 510630 China; ^4^ College of Chemistry and Materials Science Jinan University Guangzhou 510632 China

**Keywords:** immunotherapy, myeloid‐derived suppressive cells (MDSCs), multifunctional nanodrug, photodynamic therapy, PI3K*γ* inhibitor

## Abstract

An ideal tumor treatment is supposed to eliminate the primary tumor and simultaneously trigger the host antitumor immune responses to prevent tumor recurrence and metastasis. Herein, a liposome encapsulating phosphoinositide 3‐kinase gamma (PI3K*γ*) inhibitor IPI‐549 and photosensitizer chlorin e6 (Ce6), denoted by LIC, is prepared for colon cancer treatment. LIC internalized into CT26 cells generates reactive oxygen species (ROS) under laser irradiation to cause immunogenic tumor cell death, during which immunostimulatory signals such as calreticulin are released to further induce T lymphocyte‐mediated tumor cell killing. Meanwhile, IPI‐549 transported by liposome can inhibit PI3K*γ* in the myeloid‐derived suppressive cells (MDSCs), resulting in downregulation of arginase 1 (Arg‐1) and ROS to promote MDSCs apoptosis and reduce their immunosuppressive activity to CD8^+^ T cells. LIC‐mediated immunogenic photodynamic therapy synergizes with MDSCs‐targeting immunotherapy, which significantly inhibits tumor growth via facilitating the dendritic cell maturation and tumor infiltration of CD8^+^ T cells while decreasing the tumor infiltration of immunosuppressive regulatory T cells, MDSCs, and M2‐like tumor‐associated macrophages. Moreover, the synergistic therapy increases the number of effector memory T cells (T_EM_) in spleen, which suggests a favorable immune memory to prevent tumor recurrence and metastasis. The Ce6 and IPI‐549‐coloaded multifunctional nanodrug demonstrates high efficacy in colon cancer treatment.

## Graphical abstract

A liposome encapsulating phosphoinositide 3‐kinase gamma inhibitor IPI‐549 and photosensitizer chlorin e6 (Ce6) mediates immunogenic photodynamic therapy synergizing with myeloid‐derived suppressive cells (MDSCs)‐targeting immunotherapy, which significantly inhibits colon cancer growth via facilitating the dendritic cells maturation and tumor infiltration of CD8^+^ T cells while decreasing the tumor infiltration of immunosuppressive regulatory T cells, MDSCs, and M2‐like tumor‐associated macrophages.

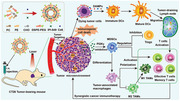

## 1 Introduction

Colon cancer is one of the most common malignant tumors derived from the digestive system.^[^ ^1^ ^]^ Recent improvements in surgical technique, chemotherapy, and employment of molecular targeted therapies have successfully improved the outcome of localized colon cancer. However, the prognosis of colon cancer patients with metastasis or postoperative recurrence still remains a great challenge.^[^ ^2^ ^]^ Notably, cancer immunotherapy which unleashes the inhibition of tumor‐mediated immunosuppressive microenvironment or stimulates the host immune system to eliminate tumor cells is emerging as a promising strategy to prevent tumor recurrence and metastasis.^[^ ^3^ ^]^ For example, immune checkpoint blockade (ICB) therapy utilizing antibodies for programmed death 1/programmed death ligand 1 (PD‐1/PD‐L1) or cytotoxic T lymphocyte‐associated protein 4 not only restores intrinsic immune response to tumors but also induces persistent antitumor immune memory after treatment.^[^ ^4^ ^]^ Other immune modulation strategies based on intra/extracellular lactic acid exhaustion or tumor microenvironment glutathione (GSH) depletion have been reported to enhance the effect of immunotherapy.^[^ ^5^, ^6^ ^]^ Whatever the specific immunotherapeutic means, tumor immune microenvironment (TIME) exerts crucial roles in modulating antitumor immunity and associates with the progression of tumors. It is well known that immune suppressive cells, including myeloid‐derived suppressive cells (MDSCs), M2‐like tumor‐associated macrophages (TAMs), and regulatory T cells (Tregs), are closely related to the suppressive TIME and responsible for tumor immune escape.^[^ ^7^ ^]^ Accumulating evidence is suggesting that MDSCs as a group of heterogeneous cells consisting of myeloid progenitor cells and immature granulocytes, immature macrophages, and immature dendritic cells (DCs) may serve as a negative regulator of antitumor immune response by suppressing various T cells functions, thereby favoring the polarization of TAMs to the protumoral M2‐like phenotype and promoting Tregs expansion.^[^ ^8^ ^]^ It is also found that phosphoinositide 3‐kinase gamma (PI3K*γ*) signaling plays a crucial role in the activation and migration of myeloid cells.^[^ ^9^ ^]^ and the immunosuppressive tumor microenvironment mediated by PI3K*γ* expressed in MDSCs facilitates tumor growth.^[^ ^10^ ^]^ IPI‐549, a selective PI3K*γ* inhibitor, may inhibit the immune response of MDSCs and reshapes TIME by reprogramming polarization of TAMs to tumoricidal M1‐like phenotype and promoting proliferation and activation of cytotoxic T lymphocytes (CTLs).^[^ ^11^ ^]^ Besides, IPI‐549 can attenuate ICB resistance and restore ICB sensitivity in cancer immumotherapy.^[^ ^12^ ^]^ Owing to its good antitumor efficacy and favorable biosafety, IPI‐549 has been promoted to a phase II clinical trial (NCT03795610).

On the other hand, photodynamic therapy (PDT) is a promising strategy for clinical cancer treatment featuring high spatiotemporal selectivity, good safety, and noninvasiveness.^[^ ^13^ ^]^ In PDT, a nontoxic photosensitizer in combination with oxygen exposed to light with a specific wavelength can generate cytotoxic reactive oxygen species (ROS) which induces apoptosis and necrosis of tumor cells and damages the tumor‐associated vasculature.^[^ ^14^ ^]^ Besides, ROS can trigger immunogenic cell death (ICD) to generate tumor‐associated antigens and release damage‐associated molecular patterns (DAMPs), including calreticulin (CRT), high‐mobility group box 1 (HMGB1), and adenosine triphosphate (ATP) from dying tumor cells.^[^ ^15^ ^]^ These DAMPs subsequently induce mature of DCs and activation of specific effector T cells and natural killer (NK) cells, thus enhancing host antitumor immune response for both primary and metastatic tumors.^[^ ^16^, ^17^ ^]^

**Scheme 1:**
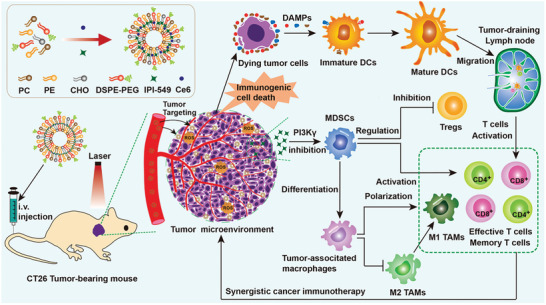
Schematic illustration of the preparation of multifunctional nanodrug using liposome to coencapsulate a photosensitizer Ce6 and a PI3K*γ* inhibitor IPI‐549 (denoted by LIC) and the LIC‐mediated colon cancer inhibition by synergistic immunogenic photodynamic therapy and MDSCs‐targeting immunotherapy.

So far, immunotherapeutics based on nanoscale delivery systems have been developed to enhance the efficacy of cancer immunotherapy.^[^ ^18^ ^]^ Specifically, nanodrug‐mediated cancer immunotherapy is achieved via targeting cancer cells to trigger ICD and targeting TIME to reverse immunosuppressive microenvironment or targeting the peripheral immune system to facilitate the generation of antigen‐presenting cells (APCs) and CTLs.^[^ ^19^ ^]^ In the present study, we constructed an immunoregulatory nanodrug, which was named LIC, by utilizing liposome to coencapsulate the ICD‐inducer chlorin e6 (Ce6) and TIME‐regulator IPI‐549. The nanoscale LIC was expected to accumulate in colon cancer to induce immunogenic ICD and target MDSC in TIME to reverse the immunosuppressive microenvironment, thereby boosting the cancer immunotherapy (**Scheme** **1**). Both in vitro and in vivo experiments were performed to explore the LIC‐mediated synergistic effect of immunogenic PDT and MDSCs‐targeting immunotherapy in colon cancer treatment.

## 2 Results and Discussion

### 2.1 Preparation and Characterization of Nanodrugs

**Figure 1:**
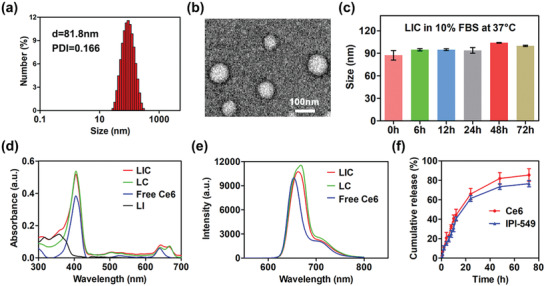
Characterization of nanodrugs. a) The size distribution of LIC. b) Transmission electron microscope (TEM) image of LIC. c) Stability of LIC in phosphate buffer saline (PBS) containing 10% FBS at 37 °C (*n* = 3). d) UV–vis absorption spectra of LIC, LC, free Ce6, and LI. e) Fluorescence spectra of LIC, LC, and free Ce6. f) Cumulative release of Ce6 and IPI‐549 from LIC at pH 6.5 (*n* = 3).

Lecithin (PC), phosphatidylethanolamine (PE), cholesterol (CHO), and 1,2‐distearoyl‐sn‐glycero‐3‐phosphoe‐thanolamine‐*N*‐[methoxy(polyethylene glycol)‐2000 (DSPE‐PEG2000) were utilized to form the liposome, and PI3K*γ* inhibitor IPI‐549 and photosensitizer Ce6 were coloaded into liposome to obtain the nanodrug (LIC) as described in the Experimental Section. The Ce6‐encapsulated liposome (LC) and IPI‐549‐encapsulated liposome (LI) were prepared for control experiments. As measured by dynamic light scattering (DLS), LIC showed an average diameter of 83.4 ± 1.6 nm and a Zeta potential of −44.4 ± 7.15 mV (**Figure** **1**a and Figure S1a, Supporting Information). Under transmission electron microscopic (TEM) observation, LIC exhibited spherical morphology with uniform size distribution (Figure 1b). The size, polydispersity index, and Zeta potential of LI and LC were similar to that of LIC (Figure S1b–d, Supporting Information). Incorporation of DSPE‐PEG2000 increased the colloidal stability and may endow the liposome with stealth property for long circulation.^[^ ^20^ ^]^ LIC in 10% fetal bovine serum (FBS)‐containing phosphate buffer saline (PBS) showed no change in size over 72 h at 37 °C, which indicated a high stability in normal physiological condition (Figure 1c). The loading contents of Ce6 and IPI‐549 in LIC were 0.95% and 3.81%, respectively. With Ce6 encapsulation, LC and LIC showed UV–vis absorption peaks at 405 and 640 nm, which were consistent with that of free Ce6 (Figure 1d). The fluorescence emission peak of LC and LIC was shown at 663 nm, exhibiting a slight redshift from 653 nm of free Ce6 (Figure 1e). These results suggested a successful loading of Ce6 into liposomes. The release behaviors of Ce6 and IPI‐549 from LIC were determined at pH 6.5. As shown in Figure 1f, 44% Ce6 and 40% IPI‐549 were released at 12 h, while 66% Ce6 and 61% IPI‐549 were released at 24 h.

### 2.2 In Vitro Cytotoxicity

**Figure 2:**
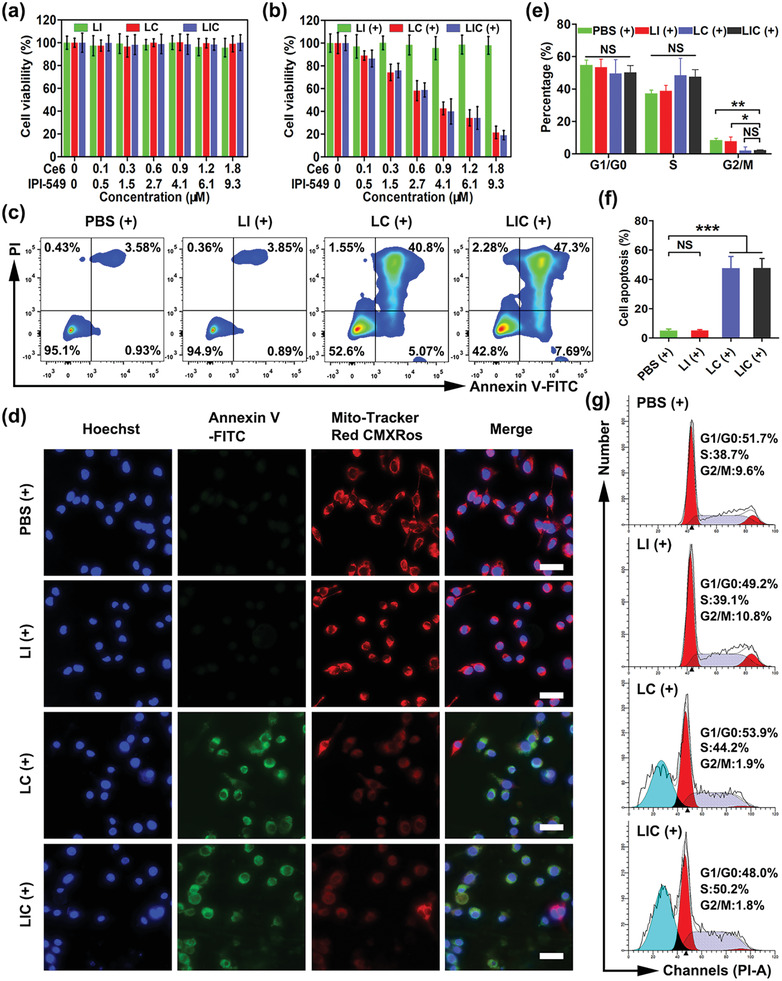
In vitro killing effects of nanodrugs on CT26 cells. In vitro cytotoxicity of LI, LC, and LIC a) without or b) with light irradiation (660 nm, 0.8 W cm^−2^, 1 min) (*n* = 4). c) CT26 cell apoptosis determined by flow cytometry. d) Fluorescence images of Annexin‐V staining (green) and staining for change in mitochondrial membrane potential (red). Scale bar: 25 µm. e) Statistical analysis of cell cycle distribution and f) apoptotic rate of CT26 cells in different groups (*n* = 3). g) Representative plots of CT26 cell cycle distribution. (+) represents 660 nm (0.8 W cm^−2^, 1 min) light irradiation. *p < 0.05, **p < 0.01, ***p < 0.001, NS: no significant difference.

To explore the cytotoxicities of nanodrugs, CT26 cells were incubated at different concentrations of LI, LC, and LIC with or without laser exposure. Without the irradiation of 660 nm laser, cells incubated at different concentrations of nanodrugs showed no obvious changes in viabilities (**Figure** **2**a). With laser irradiation, LC and LIC presented obvious cytotoxicity in a concentration‐dependent manner in CT26 cells. Both of them showed a 50% inhibitory concentration (IC50) at ≈0.7 × 10^−6^ m Ce6 (Figure 2b), which indicated that ROS generation during PDT procedure killed tumor cells as reported.^[^ ^14^, ^21^ ^]^ However, LI displayed no obvious cytotoxicity in CT26 cells, which was likely due to the lack of PI3K*γ*‐AKT signals in CT26 cells. Then, we investigated the effects of nanodrugs on CT26 cell apoptosis. Without laser irradiation, apoptotic rates of CT26 cells incubated with PBS, LI, LC, and LIC were 3.96%, 3.73%, 4.79%, and 4.15%, respectively (Figure S2a, Supporting Information). In contrast, the apoptotic rates of CT26 cells incubated with PBS, LI, LC, and LIC under light irradiation increased to 4.51%, 4.74%, 45.87%, and 54.99%, respectively (Figure 2c). It is well known that ROS‐mediated oxidative stress can lead to a decrease in mitochondrial membrane potential for cell apoptosis characterized by phosphatidylserine (PS) exposure on cell membrane.^[^ ^22^ ^]^ Here, a kit with Mito‐Tracker Red CMXRos and Annexin V‐fluorescein isothiocyanate (FITC) for apoptosis detection was employed to verify the CT26 cell apoptosis under confocal laser scanning microscope (CLSM). Cells in the PBS (+) and LI (+) groups showed normal mitochondrial membrane potential without PS exposure on the cell membrane, which were also observed for cells treated with PBS, LI, LC, or LIC without laser irradiation (Figure 2d and Figure S2e, Supporting Information). On the contrary, the LC (+) treatment and LIC (+) treatment induced obvious decrease in mitochondrial membrane potential and PS exposure (Figure 2d). These results suggested that LI treatment could not trigger obvious apoptosis of CT26 cells regardless of laser irradiation, whereas the LC treatment and LIC treatment under light irradiation significantly elevated apoptotic rates of CT26 cells (Figure 2f and Figure S2c, Supporting Information). The influence of nanodrugs on the cell cycle was further investigated. The LC and LIC treatments displayed little effect on the cell cycle of CT26 cells without light irradiation but blocked the cell cycle in the S phase to show a significant decrease of proportions of the G2/M phase with light irradiation (Figure 2e,g). Furthermore, PBS and LI showed no obvious effect on the cell cycle of CT26 cells regardless of laser irradiation (Figure S2b,d, Supporting Information). These findings confirmed that LC (+) and LIC (+) could exert phototoxicity to induce CT26 cell apoptosis and block their cell cycle, whereas LI could not kill CT26 cells directly.

### 2.3 PDT‐Elicited ICD In Vitro

**Figure 3:**
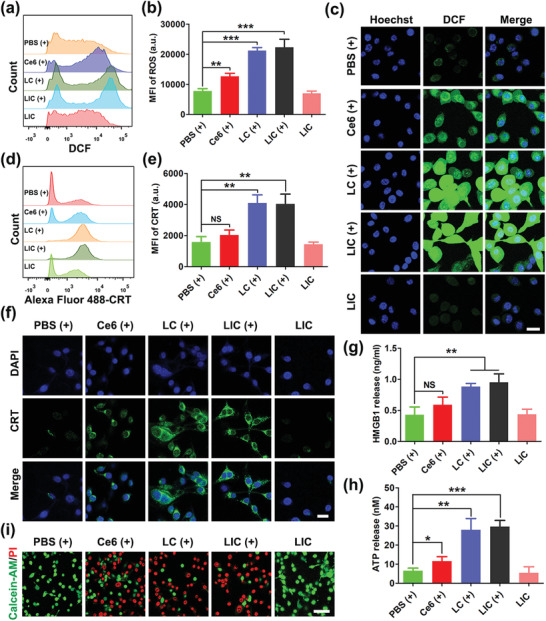
PDT‐mediated cytotoxicity and immunogenic cell death (ICD) in CT26 cells in vitro. a) Flow cytometry detection of ROS production in CT26 cells after various treatments. b) Statistical analysis of ROS generation (*n* = 3). c) Confocal imaging of ROS (green) production in CT26 cells after different treatments. Scale bar: 25 µm. d) Flow cytometry detection of ICD signal CRT expression on CT26 cells after various treatments. e) Statistical analysis of CRT expression (*n* = 3). f) Confocal imaging of CRT (green) expression on the surface of CT26 cells after different treatments. Scale bar: 25 µm. g) Determination of HMGB1 release after different treatments (*n* = 3). h) Detection of ATP secretion after different treatments (*n* = 3). i) CLSM images of live (green) and dead (red) CT26 cells after various treatments. Scale bar: 50 µm. (+) indicates 660 nm (0.8 W cm^−2^, 1 min) light irradiation. **p < 0.01, ***p < 0.001, NS: no significant difference.

PDT may produce reactive oxygen species to trigger immunogenic cell death, causing the release of damage‐associated molecular patterns (DAMPs) such as CRT, HMGB1, and ATP.^[^ ^14^, ^15^ ^]^ The ROS generation in CT26 cells receiving treatments of various nanodrugs was explored via flow cytometry analysis and CLSM observation. Under laser exposure, CT26 cells treated with LC and LIC showed remarkably enhanced mean fluorescence intensity of ROS probe 2',7'‐ Dichlorodihydrofluorescein diacetate (DCFH‐DA) in comparison with cells incubated with PBS or free Ce6 (**Figure** **3**a,b). Consistently, CT26 cells treated with LC or LIC under laser irradiation showed stronger fluorescence of ROS probe than cells receiving other treatments (Figure 3c). CRT translocation from the endoplasmic reticulum to the surface of dying cells during PDT procedure can stimulate DC mature and activation of specific effector T cells to result in enhancement of host antitumor immune response.^[^ ^16^, ^23^ ^]^ Then, we investigated whether LIC‐based PDT can induce exposure of CRT in dying tumor cells. Both flow cytometry analysis and CLSM observation confirmed that LC or LIC treatment with laser irradiation triggered exposure of CRT on CT26 cells. In comparison, PBS or free Ce6 treatment under laser irradiation induced much lower levels of CRT expression on the surface of CT26 cells (Figure 3d–f). Extracellular HMGB1 release and ATP secretion from dying tumor cells during PDT procedure can also induce host antitumor immunity.^[^ ^15^ ^]^ Here, HMGB1 and ATP secretions were measured using enzyme‐linked immunosorbent assay (ELISA). The LC (+) treatment and LIC (+) treatment showed much higher levels of HMGB1 release and ATP secretion than other treatments, which was in line with CRT expression (Figure 3g,h). Live and dead cell staining was employed to visualize the killing efficacy of PDT in CT26 cells. Under CLSM observation, cells treated with free Ce6 (+), LC (+), and LIC (+) showed much stronger red fluorescence (dead cells) and weaker green fluorescence (live cells) than cells treated with PBS (+) and LIC. Interestingly, Ce6‐encapsulated nanodrug seemed to show an enhanced killing efficacy as compared with free Ce6. That is, cells treated with LC (+) and LIC (+) even showed slightly weaker green fluorescence than cells treated with free Ce6 (+) (Figure 3i). These results suggested that the LC and LIC treatments with laser irradiation can enhance ROS release and effectively induce ICD signals in CT26 colon cancer cells in vitro.

### 2.4 Inhibition of MDSCs via Targeting PI3K*γ*‐AKT Signaling Pathway with LIC

**Figure 4:**
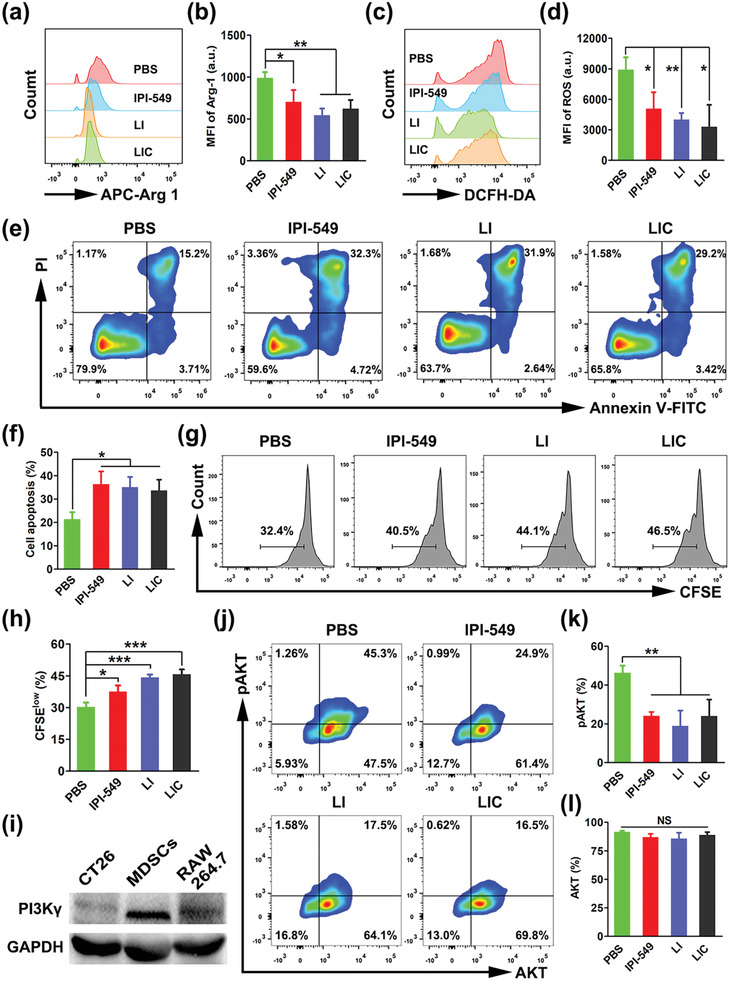
Suppression of MDSCs through PI3K*γ*‐AKT signaling pathway inhibition with IPI‐549‐based nanodrugs in vitro. a) Arg‐1 expression determined by flow cytometry and b) statistical analysis of proportions of Arg‐1 expression in MDSCs receiving various treatments (*n* = 3). c) ROS levels determined by flow cytometry and d) statistical analysis of proportions of ROS levels in MDSCs receiving various treatments (*n* = 3). e) Cell apoptosis determined by flow cytometry and f) statistical analysis of apoptotic rates in MDSCs receiving different treatments (*n* = 3). g) Proliferation of CD8^+^ T cells determined by flow cytometry analysis and h) statistical analysis of proliferation rates of CD8^+^ T cells after being cultured together with nanodrug‐pretreated MDSCs (*n* = 3). i) PI3K*γ* expression determined by western blot analysis. j) Quantification of pAKT and total AKT expressions by flow cytometry and k,l) statistical analysis of percentages of pAKT and total AKT expression in MDSCs receiving various treatments (*n* = 3). *p < 0.05, **p < 0.01, ***p < 0.001, NS: no significant difference.

The PI3K*γ*‐AKT pathway plays vital roles in cell metabolism, survival, and proliferation.^[^ ^24^ ^]^ PI3K*γ* as one of the four isoforms of PI3K family is highly expressed in myeloid cells to selectively regulate their immunosuppressive behavior.^[^ ^25^, ^26^ ^]^ It has been reported that the number of MDSCs was increased in the blood and spleen of tumor‐bearing host to promote tumor progression and metastasis.^[^ ^8^, ^27^ ^]^ The MDSCs‐mediated inhibition of T cell function is associated with l‐arginine catabolism, and l‐arginine shortage suppresses the proliferation of T cells. Meanwhile, it has been ascertained that upregulation of arginase 1 (Arg‐1) and inducible nitric oxide synthase (iNOS) in MDSCs promotes l‐arginine catabolism to suppress T cell immune response.^[^ ^8^, ^28^, ^29^ ^]^ In addition, ROS is another mediator for MDSCs to exert immunosuppressive activity. Previous studies have shown that suppression of ROS production in MDSCs can eliminate MDSCs‐mediated immunosuppressive effect in vitro.^[^ ^8^, ^30^ ^]^ To investigate the effect of LIC‐mediated PI3K*γ*‐AKT signal inhibition on the immunosuppressive activities of MDSCs in vitro, spleen‐derived MDSCs were purified by MDSC isolation kit and more than 95% of sorted cells were identified as CD11b^+^Gr‐1^+^ MDSCs (Figure S3a,c, Supporting Information). As shown in **Figure** **4**a–d, flow cytometry analysis showed that PI3K*γ* inhibition using IPI‐549‐loaded nanodrugs significantly decreased the Arg‐1 and ROS levels in MDSCs as free IPI‐549 did. Interestingly, the LI and LIC treatments even showed slightly enhanced effect to lower the Arg‐1 expression and ROS production in MDSCs as compared to free IPI‐549 treatment. Furthermore, treatments with IPI‐549, LI and LIC all remarkably increased the apoptosis of MDSCs, which was apparently due to the suppression effect of IPI‐549 on the PI3K*γ*‐AKT signaling pathway (Figure 4e,f). Then, we further tested whether the IPI‐549‐loaded nanodrugs could alleviate the suppressive activity of MDSCs on CD8^+^ T cells in vitro. CD8^+^ T cells were purified from the spleen of normal mice and their purity were ≈97% (Figure S3b,c, Supporting Information). Much higher CD8^+^ T cell proliferation was detected when they were cocultured with MDSCs pretreated with LI and LIC rather than free IPI‐549 or PBS (Figure 4g,h). As shown in Figure 4i–l, PI3K*γ* was highly expressed in purified MDSCs and inhibiting PI3K*γ* in MDSCs with free IPI‐549, LI, and LIC obviously downregulated the downstream phosphorylated AKT (pAKT), although the total AKT showed no significant difference in different treatment groups. These results strongly demonstrated that inhibiting PI3K*γ*‐AKT signal with IPI‐549‐loaded nanodrugs could effectively decrease the Arg‐1 and ROS levels in MDSCs, promote the MDSCs apoptosis, and subsequently alleviate their suppressive effects on CD8^+^ T cells. Consequently, the antitumor immune responses may be enhanced by this MDSCs‐targeting treatment.

### 2.5 Tumor‐Targeting and Biodistribution of LIC In Vivo

**Figure 5:**
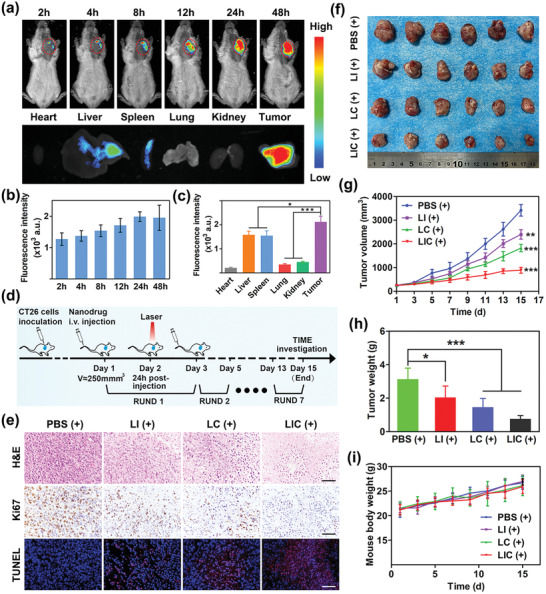
In vivo fluorescence imaging and synergistic antitumor therapy of nanodrugs. a) In vivo fluorescence imaging of CT26 tumor‐bearing mice at different time points after tail vein injection of DiR‐labeled LIC and ex vivo fluorescence imaging of main organs and tumor at 48 h after injection. b) Quantification of in vivo fluorescence intensity of tumors at different time points after injection (*n* = 3). c) Comparison of fluorescence intensities between major organs and tumor excised at 48 h after injection (*n* = 3). d) Schematic illustration of therapeutic strategy in animal model. e) Photograph of tumors excised from mice receiving various treatments. Scale bar: 50 µm. g) Tumor growth curves for mice receiving different treatments (*n* = 6). Data are shown as mean ± standard error of mean (SEM). h) Tumor weights in different therapeutic groups and i) changes of body weight during various treatments (*n* = 6). *p < 0.05, **p < 0.01, ***p < 0.001.

Nanodrugs have been reported to accumulate in solid tumors via passing through the interendothelial gaps of tumor blood vessels or actively passing through endothelial cells.^[^ ^31^, ^32^ ^]^ Fluorescence imaging study was performed to evaluate the tumor‐targeting and distribution of LIC in CT26 tumor‐bearing mice. LIC was labeled with fluorescent dye 1,1‐dioctadecyl‐3,3,3,3‐tetramethylindotricarbocyanine iodide (DiR) to make the nanodrug imaging‐visible. The fluorescence intensity of DiR‐labeled LIC gradually increased in tumor tissues to reach the highest at 24 h after tail vein administration (**Figure** **5**a,b), which indicated that 24 h postadministration was a proper time point to perform laser irradiation in the antitumor PDT experiments. Further, the fluorescence signal of DiR‐labeled LIC remained strong at 48 h postadministration. These results indicated that LIC could effectively accumulate in the CT26 tumor in vivo. At 48 h after injecting the DiR‐labeled LIC, the ex vitro fluorescence intensity of tumor tissues were much higher than that of main organs including heart, liver, spleen, lung, and kidney (Figure 5c), which suggested a favorable accumulation of LIC in the CT26 tumor essential for a targeted regulation of tumor immune microenvironment. After intravenous injection, nanoparticles may be covered by serum proteins and form a protein corona to cause a uptake of nanoparticles by the mononuclear phagocyte system (MPS).^[^ ^33^ ^]^ Generally, liver and spleen have abundant MPS which can arrest the circulating nanoparticles. Thus, imaging‐visible LIC showed fluorescence signal distribution in spleen in ex vivo fluorescence imaging, which was also observed in other studies.^[^ ^17^, ^34^ ^]^

### 2.6 Antitumor Effects In Vivo

It has been reported that IPI‐549 may inhibit PI3K*γ* in MDSC to reverse the immunosuppressive tumor microenvironment for an antitumor immunotherapy,^[^ ^10^, ^11^ ^]^ and Ce6 under laser irradiation may induce ICD of tumor cells to release immunogenic signals boosting the anticancer immunotherapy.^[^ ^19^ ^]^ The therapeutic schedule illustrated in Figure 5d was carried out to explore the synergistic antitumor potential of the codelivered Ce6 and IPI‐549. Tumors were excised from tumor‐bearing mice at the end of treatment (Figure 5f). Tumor growth was quick in animals treated with PBS (+), showing tumor volumes over 3000 mm^3^ at 15 d post‐treatment. Treatments with different nanodrugs all showed obvious inhibitory effects on tumor growth. Moreover, in comparison with the LI (+) or LC (+) treatments, LIC (+) treatment exhibited the best effect to inhibit tumor growth, clearly indicating that Ce6 and IPI‐549 acted synergistically to enhance antitumor immunotherapy (Figure 5g). Consistently, tumor weight in the LIC (+) treatment group was much lower than that in the LI (+) treatment group and LC (+) treatment group (Figure 5h). The LI treatment and LI (+) treatments resulted in similar tumor growth and weight, which suggested that the laser irradiation did not enhance the antitumor effect of nanodrug only incorporating IPI‐549 (Figure S4a–c, Supporting Information). To assess the effects of nanodrugs on tumor proliferation, apoptosis and necrosis, tumor sections in each group were subjected to hematoxylin‐eosin (H&E), Ki67, and terminal deoxynucleotidyl transferase‐mediated dUTP‐biotin nick end‐labeling (TUNEL) staining. As shown in Figure 5e, tumor tissue from the animal groups treated with LIC (+) displayed the highest apoptosis rate and the lowest proliferation rate. Obviously, although the LI (+)‐mediated immunotherapy and LC‐mediated immunogenic PDT were both effective to inhibit tumor growth, treatment with LIC (+) further enhanced the therapeutic effect due to the synergistic effects of IPI‐549 and Ce6.

Aiming to evaluate the side effects of nanodrugs in vivo, body weight of mice were recorded during the treatment, and blood and main organs of mice were collected at the end of treatments for further analysis. No significant body weight loss was found (Figure 5i), and no obvious pathological alterations in the main organs (heart, liver, spleen, lung, and kidney) were observed for all treatment groups (Figure S5a, Supporting Information). Serum markers of liver function (aspertate aminotransferase (AST), alanine aminotransferase (ALT)) and renal function (blood urea nitrogen (BUN), creatinine (Cr)) were at normal levels and showed no significant differences between the four groups (Figure S5b–d, Supporting Information). These results confirmed that LIC had low side effects in vivo.

### 2.7 Nanodrugs‐Mediated TIME Remodeling for Immunotherapy

To better understand why the LIC (+) treatment showed much better tumor growth inhibition than the LC (+) treatment and LI (+) treatment, we further examined the infiltration of various immune cells in tumor tissues and spleens, including MDSCs, T‐cell subpopulations, TAMs, DCs, and NK cells. Gating strategies to investigate percentages of various immune cells in tumor and spleen were shown in Figure S6 in the Supporting Information.

**Figure 6:**
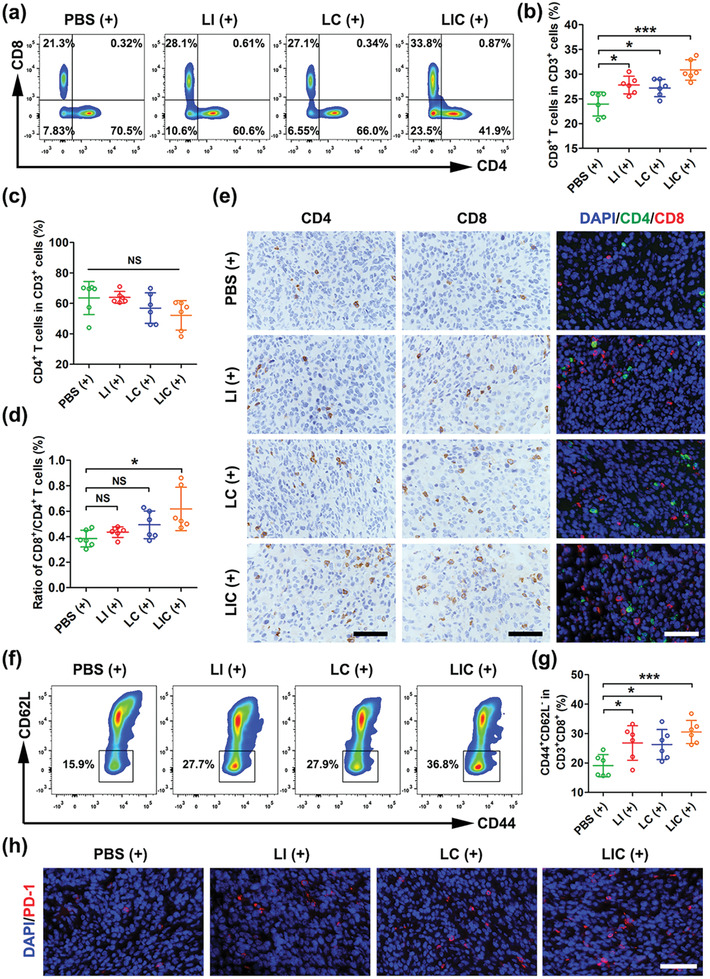
Nanodrugs enhanced antitumor immune responses in CT26 tumor. a) CD8^+^ T and CD4^+^ T cells ratios in tumor tissues determined by flow cytometry after different treatments (gated on CD3^+^ cells). b) Statistical analysis of the ratio of CD8^+^ T cells or c) the ratio of the CD4^+^ T cells to the CD3^+^ cells in different groups (*n* = 6). d) Statistical analysis of the ratio of CD8^+^ T cells to CD4^+^ T cells in different groups (*n* = 6). e) Immunohistochemical and immunofluorescent staining of CD4 and CD8 in CT26 tumors after different treatments. Scale bar: 50 µm. f) Representative plots of effector memory T (T_EM_) cells in spleen after different treatments determined by flow cytometry (gated on CD3^+^CD8^+^ T cells). g) Statistical analysis of T_EM_ proportions in CD3^+^CD8^+^ T cells in different groups (*n* = 6). h) Immunofluorescent staining of the nucleus (blue) and PD‐1 expression (red) in CT26 tumors in different groups. Scale bar: 50 µm. *p < 0.05, ***p < 0.001, NS: no significant difference.

It is well known that effector T cells and regulatory T cells are the main T cell subpopulations involving in tumor immune response, PDT‐induced ICD may facilitates T cell recruitment and activation in tumor tissues to increase antitumor immunity.^[^ ^14^, ^35^ ^]^ CD8^+^ T cells are the major subsets that exert crucial roles in antitumor immunotherapy by producing several cytokines like interferon‐γ (IFN‐γ) , granzyme, and perforin.^[^ ^36^ ^]^ On the other hand, Arg‐1, ROS, and iNOS produced by MDSCs are mainly responsible for tumor growth and spreading as they restrain the CD4^+^ and CD8^+^ T cells immune response.^[^ ^28^ ^]^ Therefore, the synergistic effect of PDT and MDSCs‐targeting immunotherapy of IPI‐549 on major T cell subsets were evaluated. As shown in **Figure** **6**a,b, the ratio of CD8^+^ T cells to CD3^+^ cells were significantly increased in the LI (+) and LC (+) treatment groups as compared with the PBS (+) treatment group. Moreover, the LIC (+) treatment enhanced tumor infiltration of CD8^+^ T cells more effectively than the LI (+) treatment and LC (+) treatment. CD4^+^ T and CD8^+^ T cells are main subpopulations of CD3^+^ T cells. Although it seemed that the percentage of CD4^+^ T cells in CD3^+^ T cells in the LIC (+) treatment group showed a slight decrease, the difference between the four groups had no statistical difference (Figure 6c). In addition, an increased ratio of CD8^+^ cells to CD4^+^ T cells in the LIC (+) treatment group was also observed (Figure 6d). The immunohistochemical (IHC) and immunofluorescent staining for CT26 tumor sections indicated the increased infiltration of CD8^+^ T cells in tumor tissues in the three nanodrug treatment groups (Figure 6e). From these results, the increased tumor infiltration of CD8^+^ T cells was a major cause for the better tumor killing of LIC (+) treatment.

Admittedly, MDSCs promote the expansion of regulatory T cells (Tregs) to maintain an immunosuppressive TIME.^[^ ^8^, ^37^ ^]^ However, immunogenic PDT could decrease the number of Tregs in tumor tissues to remodel the immunosuppressive TIME.^[^ ^38^ ^]^ As shown in Figure S7a,b in the Supporting Information, staining of Foxp3 as biomarker for Tregs indicated that the numbers of Tregs in tumor tissue were significantly decreased in the LI (+) treatment group and the LIC (+) treatment group and no obvious decrease of Tregs was observed for the LC (+) treatment group as compared to the PBS treatment group (*p* > 0.05).

Antitumor immune memory is essential for a long‐term prevention of tumor recurrence and metastasis. Memory T cells are broadly subdivided into central memory T cells (T_CM_) and effector memory T cells (T_EM_). T_CM_ mainly migrates to lymphoid tissues whereas T_EM_ can traffic to lymphoid tissues and peripheral nonlymphoid tissues.^[^ ^39^, ^40^ ^]^ Notably, T_EM_ can generate some critical cytokines like tumor necrosis factor‐α (TNF‐*α*) and INF‐*γ* to trigger a strong protective immunological memory when it encounters the same tumor‐specific antigens.^[^ ^41^ ^]^ To investigate the immune memory effect of the nanodrugs, CD8^+^ T_EM_ and T_CM_ in spleen were analyzed by flow cytometry. Compared with the PBS (+) treatment group, the LI (+) treatment group and the LC (+) treatment group showed much higher percentages of T_EM_ (CD3^+^CD8^+^CD44^+^CD62L^−^) in spleen. More excitingly, the synergistic therapy using LIC (+) induced the highest T_EM_ proportion in spleen (Figure 6f,g). The percentage of T_CM_ (CD3^+^CD8^+^CD44^+^CD62L^+^) in the LIC (+) treatment group was much lower than that in other groups, which may be attributed to the differentiation of T_CM_ to T_EM_ stimulated by tumor‐specific antigen (Figure S8, Supporting Information). These results implied that the combined therapy of LIC‐mediated PDT and MDSC‐targeted immunotherapy may possess a potential to prevent colon cancer recurrence due to its immune memory effect. Myeloid cells‐enriched tumors treated by IPI‐549 could upregulate the PD‐1 expression on CD8^+^ T cells.^[^ ^12^ ^]^ Moreover, immunogenic PDT can induce CD8^+^ T cells activation and recruitment to tumors, while PD‐1 upregulation was founded in the process of T cells activation.^[^ ^14^, ^42^ ^]^ Thus, the increased PD‐1 expression in the LIC (+) treatment group observed in our study is likely due to the IPI‐549‐mediated PD‐1 upregulation and the activation of CD8^+^ T cells induced by the immunogenic PDT (Figure 6h and Figure S9, Supporting Information).

**Figure 7:**
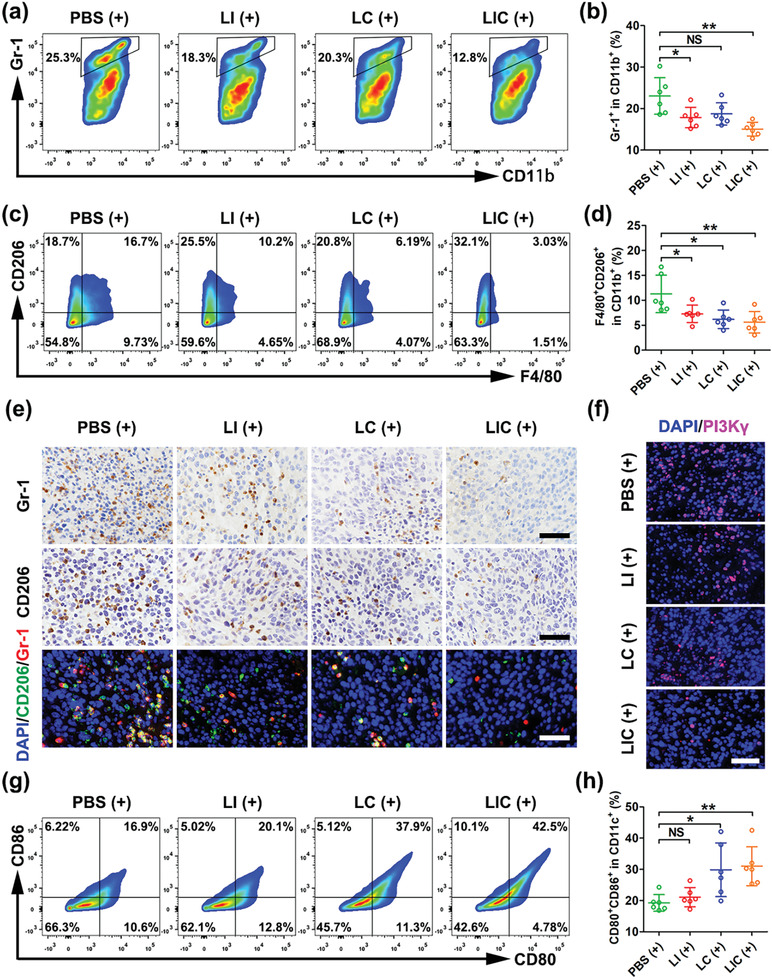
Nanodrugs regulated myeloid cells to enhance antitumor immune responses in CT26 tumors. a) MDSCs in tumor tissues after different treatments determined by flow cytometry (gated on CD11b^+^ cells). b) Statistical analysis of MDSCs (CD11b^+^Gr‐1^+^) ratio in CD11b^+^ cells in different treatment groups (*n* = 6). c) M2‐like TAMs in tumor tissues after various treatments determined by flow cytometry (gated on CD11b^+^ cells). d) Statistical analysis of M2‐like TAMs (CD11b^+^F4/80^+^CD206^+^) ratio in CD11b^+^ cells (*n* = 6). e) Immunohistochemical and immunofluorescent staining of Gr‐1 and CD206 in CT26 tumors after different treatments. Scale bar: 50 µm. f) Fluorescence images of nucleus (blue) and PI3K*γ* (red) expression in CT26 tumors after different treatments. Scale bar: 50 µm. g) Mature DCs in tumor tissues after various treatments determined by flow cytometry (gated on CD11c^+^ cells). h) Statistical analysis of mature DCs (CD80^+^CD86^+^) ratio in CD11b^+^ cells (*n* = 6). *p < 0.05, **p < 0.01, NS: no significant difference.

Previous studies have revealed that PI3K*γ* overexpressed in MDSCs plays key roles in the regulation of immunosuppressive tumor microenvironment (TME) during tumor growth and suppression of PI3K*γ* can reshape tumor immune microenvironment to inhibit tumor growth.^[^ ^10^ ^]^ Here, we found that the LI (+)‐treated mice showed a decreased number of MDSCs in tumor tissues, and the LIC (+)‐treated mice further displayed a remarkable decrease of MDSCs, apparently owing to a synergistic effect of IPI‐549 and PDT as determined by flow cytometry analysis (**Figure** **7**a,b,e). These results were in line with previous reports that immunogenic PDT procedure could reduce infiltration of MDSCs in tumor tissues.^[^ ^35^ ^]^ Consistently, immunofluorescent staining showed that the LIC (+) treatment significantly downregulated the expression of PI3K*γ* (Figure 7f and Figure S10, Supporting Information). Obviously, the LIC (+) treatment effectively reduced the number of MDSCs in tumors, which was favorable for tumor growth inhibition.

MDSCs have the potential to differentiate to TAMs in the tumor microenvironment, and inhibition of PI3K*γ* on MDSCs promotes polarization of TAMs from the protumoral M2‐like phenotype to the antitumoral M1‐like phenotype.^[^ ^11^, ^43^, ^44^ ^]^ As shown in Figure 7c–e, the LI (+) and LC (+) treatments resulted in a significant decrease of M2‐like TAMs (CD11b^+^F4/80^+^CD206^+^) and the LIC (+) treatment further reduced the infiltration of M2‐like TAMs in tumor tissue. In addition, flow cytometry analysis showed a much higher percentage of M1‐like TAMs (CD11b^+^F4/80^+^CD16/32^+^) in the LI (+) treatment group than the PBS (+) treatment group. Surprisingly, the percentages of M1‐like TAMs in the LC (+) and LIC (+) treatment groups were found to be decreased as compared to that in the PBS (+) treatment group, which was likely due to the damage of PDT to macrophages (Figure S11a,b, Supporting Information).

DCs maturation is a crucial step for further presentation of tumor‐specific antigens and activation of T lymphocytes.^[^ ^16^, ^45^ ^]^ It has been reported that PDT may cause CRT expression on the surface of tumor cells to act as a distinct biomarker of ICD which is an “eat me” signal to mediate the engulfment of dying cancer cells and cancer‐cell debris by immature DCs and to induce the succeeding antitumor immune response.^[^ ^46^ ^]^ Thus, CRT in tumor tissue during PDT was tested using immunofluorescence assay. As shown in Figure S12 in the Supporting Information, the LC (+) and LIC (+) treatments appeared to trigger much more CRT expression in tumor tissues than the LI (+) and PBS (+) treatments. Then, we examined the influences of various treatments on the DCs maturation. Costimulatory molecules CD80 and CD86 were regarded as biomarkers for DCs maturation. In line with the CRT expression, the percentages of mature DCs in the LC (+) and LIC (+)‐treated mice were much higher than that in the PBS (+)‐treated mice (Figure 7g,h).

An immunogenic PDT may increase the number of NK cells in tumor tissues or draining lymph nodes, which is in favor of antitumor immunity.^[^ ^47^ ^]^ However, MDSCs have been reported to inhibit the interleukin‐2 (IL‐2)‐mediated cytotoxicity of NK cells by affecting the activity of Stat5 in tumor‐bearing mice and patients.^[^ ^48^ ^]^ Here, we investigated the effect of LIC (+) therapy on NK cell activation in spleen. The percentage of NK cells in spleen was obviously increased in the LIC (+)‐treated mice as compared to other treatment groups (Figure S13, Supporting Information). Thus, the LIC nanodrug could elicit strong host antitumor immune effects in primary tumors as well as peripheral immune organs.

## 3 Conclusion

A nanodrug incorporating photosensitizer Ce6 and PI3K*γ* inhibitor IPI‐549 was prepared for the synergistic photodynamic therapy and MDSCs‐targeting immunotherapy of colon cancer. This nanodrug possessed good biocompatibility and excellent tumor‐targeting properties. Ce6 delivered to tumor could exert PDT effects under light irradiation to trigger cascade immune responses, including immunogenic signals released from tumor cells, DCs maturing, and infiltration of antitumor T‐lymphocytes in the tumor microenvironment. Meanwhile, IPI‐549 released in the tumor microenvironment could inhibit PI3K*γ* in MDSCs to promote proliferation and activation of CD8^+^ T cells, decrease Tregs, reprogram the polarization of TAMs to M1‐like phenotype. Thus, the LIC‐based immunogenic PDT and IPI‐549‐induced immunotherapy via targeting PI3K*γ*‐AKT signaling pathway in MDSCs synergistically boosted the antitumor immune effect and immune memory. In animal study, the LIC (+) treatment most significantly inhibited the CT26 tumor growth. These results highlight the great potential of this multifunctional nanodrug for a highly effective cancer treatment.

## 4 Experimental Section

### Reagents, Synthesis, and Characterization of Nanodrugs

Lecithin (PC), cholesterol (Chol), and DSPE‐PEG2000 were obtained from Advanced Vehicle Technology (shanghai, China). PE was purchased from Macklin (shanghai, China). IPI‐549 was purchased from MedChemExpress Co., Ltd (Shanghai, China). Chlorin e6 (Ce6) was purchased from J&K Scientific Co., Ltd. (Shanghai, China). All reagents utilized in current study were analytical grade. For the synthesis of nanodrug loaded with IPI‐549 and Ce6 (LIC), a total of 30 mg of PC: PE: Chol: DSPE‐PEG2000 (with a molar ratio of 50: 25: 25: 5), 1.2 mg IPI‐549, and 0.3 mg Ce6 were sufficiently dissolved in 10 mL chloroform in the dark environment. Then, a rotary evaporation procedure (42 °C, 100 rpm, 30 min) was performed to remove solvent and form lipid films. Next, 5 mL ultrapure water was added to peel off lipid films. The suitable particle size of nanodrug was obtained via a probe supersonic method (power level 130 W, 20 kHz, 20 min). Nanodrugs loaded with single IPI‐549 (LI) or Ce6 (LC) were prepared with only IPI‐549 or Ce6 addition procedure. Further, LIC encapsulated with 0.3 mg DiR was prepared for in vivo fluorescence imaging. The size distribution and morphology of the nanodrugs were measured by DLS and transmission electron microscopy (TEM). The UV–vis absorption spectra of free Ce6 and nanodrugs were obtained by UV–vis spectrometry. Further, the fluorescence spectra of free Ce6, LC, and LIC were measured by fluorescence spectroscopy at 401 nm excitation. To explore the stability of LIC, 0.1 mL nanolipsome solution was suspended in 0.9 mL PBS containing 10% FBS (pH 7.4, 37 °C). At determined time periods, the diameter of LIC was measured by DLS. To detect the loading capacity and encapsulation efficiency of LIC, a dialysis bag (35 000 Da) was utilized to remove free drugs. After ultrafiltration, nanodrugs were freeze dried to a powdery form for further detection. To explore the cumulative release of Ce6 and IPI‐549 in vitro, a 5 mL solution of LIC, filtered through a sterile filter (0.45 µm), was added to a dialysis bag (14 kDa) which were then transferred into the 10 mL release medium (PBS containing 0.5% Tween 80, pH 6.5). At determined time points, 1 mL release medium was taken out for further detection and simultaneously replaced by the same volume fresh medium. Ce6 and IPI‐549 concentrations were determined by UV spectrophotometry and high‐performance liquid chromatography, respectively.

### In Vitro Cytotoxicity Experiments

CT26 colon cancer cells were cultured in Roswell Park Memorial Institute (RPMI)‐1640 medium with 10% FBS and 1% penicillin/streptomycin at 37 °C in an atmosphere of 5% CO_2_. Cell Counting Kit‐8 (CCK‐8) assay was performed to detect cytotoxicity of nanodrugs with or without light irradiation. Briefly, CT26 cells were seeded into 96‐well plates at a density of 5 × 10^3^ cells per well and cultured for 24 h. Then, the cells were treated with various concentrations of LI, LC, and LIC for 6 h. After washed with PBS, the cells were irradiated with or without a 660 nm laser for 1 min (0.8 W cm^−2^) and cultured for another 24 h. Cell viability was assessed by CCK‐8 (Beyotime Biotechnology, China) detection according to the manufacturer's protocol.

For apoptosis analysis, CT26 cells were seeded into 24‐well plates at a density of 5 × 10^4^ cells per well and cultured for 24 h. Then, the cells were treated with PBS, LI, LC, and LIC at an identical concentration of Ce6 (0.8 × 10^−6^ m). After 6 h of incubation, the cells were washed with PBS and irradiated with or without a 660 nm laser for 1 min (0.8 W cm^−2^), followed by another 24 h incubation. These cells were harvested and stained with Annexin V‐FITC/propidium iodide (PI, keyGEN BioTECH, China) for flow cytometry analysis. For immunofluorescent imaging, the nanodrugs‐treated CT26 cells were incubated with Annexin V‐FITC/Mito‐Tracker Red CMXRos/Hoechst 33342 (keyGEN BioTECH, China) and observed by CLSM.

For cell cycle analysis, CT26 cells were seeded into 12‐well plates at a density of 2 × 10^5^ cells per well and cultured for 24 h. Then, the cells were treated with PBS, LI, LC, and LIC at an identical concentration of Ce6 (0.8 × 10^−6^ m). After 6 h of incubation, the cells were washed with PBS and irradiated with or without a 660 nm laser for 1 min (0.8 W cm^−2^), followed by another 24 h incubation. These cells were harvested and fixed in ice‐cooled 70% ethanol for 18 h. Then, the cells were stained with RNase A and PI (keyGEN BioTECH, China) for 30 min at room temperature. DNA contents were determined by flow cytometry analysis.

### In Vitro Detection of ROS Generation

For flow cytometry analysis, CT26 cells were seeded into 24‐well plates at a density of 5 × 10^4^ cells per well and cultured for 24 h. Then, the cells were treated with PBS, free Ce6, LC, and LIC at an identical concentration of Ce6 (0.8 × 10^−6^ m). After 6 h of incubation, the cells were stained with DCFH‐DA (Beyotime Biotechnology, China) for 0.5 h at 37 °C and then exposed to a 660 nm laser for 1 min (0.8 W cm^−2^). These cells were harvested and ROS generation was determined by flow cytometry analysis. For CLSM imaging, CT26 cells (1 × 10^5^ cells per dish) were seeded into a 35 mm dish with 2 mL RPMI‐1640 culture medium. PBS, Free Ce6, LC, or LIC were added to different dishes at a same Ce6 concentration (0.8 × 10^−6^ m) and incubated for 6 h. The cells were stained with DCFH‐DA and 4’,6‐diamidino‐2‐ phenylindole (DAPI) for 20 min at 37 °C and then exposed to a 660 nm laser for 1 min (0.8 W cm^−2^). Then, these cells were observed by CLSM.

### In Vitro Determination of Crucial ICD Biomarkers

For flow cytometry analysis of CRT expression, CT26 cells were seeded into 24‐well plates at a density of 5 × 10^4^ cells per well and cultured for 24 h. Then, the cells were treated with PBS, free Ce6, LC, and LIC at an identical concentration of Ce6 (0.8 × 10^−6^ m). After 6 h of incubation, the cells were exposed to a 660 nm laser for 1 min (0.8 W cm^−2^) and cultured for another 24 h. The cells were harvested and stained with the antimouse CRT antibody (Beyotime Biotechnology, China) and a second fluorescent antibody. Finally, the expression of CRT was detected by flow cytometry. For CLSM imaging, CT26 cells (1 × 10^5^ cells per dish) were seeded into a 35 mm dish with 2 mL RPMI‐1640 culture medium. PBS, Free Ce6, LC, or LIC were added to different dishes at a same Ce6 concentration (0.8 × 10^−6^ m) and incubated for 6 h. After 6 h of incubation, the cells were exposed to a 660 nm laser for 1 min (0.8 W cm^−2^) and cultured for another 24 h. After fixed with 4% paraformaldehyde for 15 min, the cells were stained with the antimouse CRT antibody (Beyotime Biotechnology, China) for 2 h and subsequently labeled with a PE‐conjugated secondary antibody for 1 h at room temperature. After stained with DAPI for 10 min, the expression of CRT was observed by CLSM.

To examine the release of HMGB1 and ATP, CT26 cells were seeded into 24‐well plates at a density of 5 × 10^4^ cells per well and then cultured for 24 h. The cells were treated with PBS, free Ce6, LC, and LIC at an identical concentration of Ce6 (0.8 × 10^−6^ m). After 6 h of incubation, the cells were exposed to a 660 nm laser for 1 min (0.8 W cm^−2^) and cultured for another 24 h. Then, the cell supernatants were collected and determined using a HMGB1 ELISA Kit or an ATP ELISA Kit, following the manufacturer's instructions.

### Live/Dead Viability Assay In Vitro

CT26 cells (2 × 10^5^ cells per dish) were seeded into a 35 mm dish with 2 mL RPMI‐1640 culture medium. PBS, Free Ce6, LC, or LIC were added to different dishes at a same Ce6 concentration (0.8 × 10^−6^ m) and incubated for 6 h. After incubation, the cells were exposed to a 660 nm laser for 1 min (0.8 W cm^−2^) and cultured for another 24 h. Then, the cells were stained with Calcein acetoxymethylester (AM) (2 × 10^−6^ m) and PI (2 × 10^−6^ m) (keyGEN BioTECH, China) and observed by CLSM.

### MDSCs and CD8^+^ T Cells Purification Experiments

Male BALB/c mice aged 4–5 weeks were inoculated with CT26 cells to obtain tumor‐bearing mice according to the protocol of animal model. Two weeks after CT26 cells inoculation, mice were sacrificed and spleens were collected to filter through a 70 µm filter to prepare a single cell suspension. Then, mononuclear cells were obtained using a lymphocyte separation medium. MDSCs were isolated using the antimouse Gr‐1‐Biotin antibody and antibiotin MicroBeads (Miltenyi Biotech, Germany) according to the protocol of the MDSC‐kit. The purity of isolated MDSCs was identified by staining with antimouse CD11b‐FITC (BioLegend, USA) and antimouse Gr‐1‐PE/Cy7 (BioLegend, USA) antibodies and further detected by flow cytometry. For CD8^+^ T cells purification, normal BALB/c mice aged 4–5 weeks were sacrificed and spleens were collected to filter through a 70 µm filter to prepare a single cell suspension. Similarly, mononuclear cells were obtained using lymphocyte separation medium and CD8^+^ T cells were directly isolated using the anti‐CD8a (Ly‐2) MicroBeads (Miltenyi Biotech, Germany) according to the protocol. The purity of isolated CD8^+^ T cells was identified by incubating with antibodies of antimouse CD3‐PerCP/Cyanine 5.5 (BioLegend, USA) and antimouse CD8‐APC (BioLegend, USA) and measured by flow cytometry analysis.

### PI3K*γ* Inhibition Studies In Vitro

Western blot analysis was performed to identify the expression of PI3K*γ* in MDSCs. First, total protein was extracted from purified MDSCs and protein contents were detected by the bicinchoninic acid (BCA) kit. After separating on a sodium dodecyl sulfate‐polyacrylamide gel electrohoresis (SDS–PAGE) gel, proteins were transferred to polyvinylidene difluoride membranes and incubated with the anti‐PI3K*γ* antibody (Abcam, USA) and subsequent a secondary antibody. Then, PI3K*γ* proteins were visualized using an electrochemiluminescence (ECL) kit. Proteins extracted from CT26 and RAW264.7 cells were used as negative and positive controls, respectively.

For experiments of PI3K*γ* inhibition in MDSCs, purified MDSCs were seeded into 24‐well plates (5 × 10^4^ cells per well) and cultured in RPMI 1640 medium with 10% FBS and 1% penicillin/streptomycin at 37 °C in an atmosphere of 5% CO_2_ and treated with PBS, free IPI‐549, LI, and LIC respectively at an identical concentration of IPI‐549 (2 µg mL^−1^) for 12 h. For Arg‐1 detection, MDSCs were inoculated with fixation and permeabilization buffer for 45 min at room temperature. Then, the MDSCs were labeled with the antimouse Arg‐1‐APC antibody (ebioscience, USA) and analyzed by flow cytometry. For ROS detection, MDSCs pretreated with nanodrugs or controls were inoculated with DCFH‐DA (Beyotime Biotechnology, China) for 20 min at 37 °C and ROS levels in MDSCs were analyzed by flow cytometry. For apoptosis detection, MDSCs pretreated with nanodrugs or controls were harvested and stained with Annexin V‐FITC/propidium iodide (keyGEN BioTECH, China) and detected by flow cytometry. For pAKT and total AKT detection, MDSCs pretreated with nanodrugs or controls were incubated with antibodies of antimouse pAKT‐FITC (ebioscience, USA) and antimouse AKT‐PE (ebioscience, USA) and measured by flow cytometry. Purified CD8^+^ T cells were labeled with carboxyfluorescein succinimidyl ester (CFSE) (5 × 10^−6^ m) and seeded into 24‐well plates (5 × 10^5^ cells per well) which were pre‐coated with 2 µg mL^−1^ anti‐CD3 antibody (clone 145‐2C11), Then, purified MDSCs pretreated by PBS, free IPI‐549, LI or LIC (IPI‐549, 2 µg mL^−1^) for 4 h were mixed with CD8^+^ T cell suspension at a ratio of 1:2. Then, 1 µg mL^−1^ anti‐CD28 antibody (clone 37.51) and 30 ng mL^−1^ IL‐2 were added in MDSCs and CD8^+^ T cells coculture system. After 64 h, the cells were harvested and stained with Ghost Dyes (Tonbo Biosciences, USA) and the antimouse CD8‐APC (BioLegend, USA) antibody in order. CFSE signal (gated on live and CD8^+^ T cells) were analyzed by flow cytometry which represented the proliferation of CD8^+^ T cells.

### Animal Model

Male BALB/c mice aged 3–5 weeks were purchased from Yancheng Biotechnology Co., Ltd (Guangzhou, China). All animal experimental procedures were approved by the Institutional Animal Care and Use Committee of the Sun Yat‐sen University. To establish a tumor model, CT26 cells (1 × 10^6^ cells per mouse) were subcutaneously implanted in the right axilla. Tumor size and mouse weight were recorded every day. The tumor volume was calculated using the following formula: length × width^2^ × 0.5.

### In Vivo Fluorescence Imaging

DiR‐labeled LIC was injected into tumor‐bearing mice via the tail vein at a DiR dose of 0.75 mg kg^−1^ body weight. Then, the fluorescence imaging system (Carestream IS 4000, USA) was performed to capture fluorescence images in vivo at determined time points after injection. At 48 h postadministration, fluorescence imaging of main organs and tumors was captured in vitro.

### In Vivo Antitumor Assays and Toxicity to Main Organs

When tumor volumes reached 250 mm^3^, tumor‐bearing mice were randomly separated into five groups (*n* = 6 per group) and injected with 250 µL of PBS, LI, LI, LC, and LIC via tail vein once every other day at IPI‐549 and Ce6 doses of 3.0 and 0.75 mg kg^−1^ body weight, respectively. Twenty four hours postinjection, tumor‐bearing mice were exposed to a 660 nm laser for 1 min (0.8 W cm^−2^). Tumor size and mouse weight were recorded every two days. When tumor growth reached ≈3000 mm^3^, the mice were sacrificed and tumors were dissected for H&E, Ki67, and TUNEL staining and TIME analysis.

To investigate the toxicity of nanodrugs in vivo, the sera of tumor‐bearing mice were obtained to evaluate liver function (AST, ALT) and renal function (CR, BUN) at the end of treatment. Simultaneously, main organs (heart, liver, spleen, lung, and kidney) were obtained from tumor‐bearing mice to perform H&E staining.

### Analysis of Host Antitumor Immune Responses

At the end of a treatment course, host antitumor immune responses were analyzed. Specifically, the mice were sacrificed and tumors, as well as spleens, were collected, digested, and prepared single‐cell suspensions. To determine T‐cell subpopulations, the suspensions of tumors were stained with antibodies of antimouse CD3‐PerCP/Cyanine 5.5, antimouse CD4‐FITC, antimouse CD8‐APC. To determine tumor‐associated macrophages (TAMs), the suspensions of tumors were labeled with antibodies of antimouse CD11b‐FITC, antimouse F4/80‐APC, antimouse CD16/32‐PerCP/Cyanine 5.5, and antimouse CD206‐BV421. To detect MDSCs, the suspensions of tumors were stained with antibodies of antimouse CD11b‐FITC and antimouse Gr‐1‐PE/Cy7. To analyze mature DCs, the suspensions of tumors were incubated with antibodies of antimouse CD11c‐PE/Cy7, antimouse CD80‐FITC, and antimouse CD86‐BV605. To evaluate the immune memory, the suspensions of spleens were incubated with antibodies of antimouse CD3‐PerCP/Cyanine 5.5, antimouse CD8‐APC, antimouse CD44‐AF700, antimouse CD62L‐PE. To identify NK cells, the suspensions of spleens were labeled with antibodies of antimouse CD3‐PerCP/Cyanine 5.5 and antimouse CD49‐APC. Finally, the populations of immune cells in suspensions were assessed using flow cytometry. All fluorescent‐labeled flow cytometric antibodies in this part were purchased from BioLegend (San Diego, CA, USA).

### Immunohistochemical and Immunofluorescence Analysis

At the end of treatments, mice were sacrificed and tumors were collected to prepare paraffin‐embedded sections. For immunohistochemical analysis, the sections were incubated with primary antibodies and secondary antibodies after deparaffinating, quenching of endogenous peroxidase activity and blocking with bovine serum albumin (BSA). Then, the sections were scanned with an optical microscope and interesting areas were captured. For immunofluorescence analysis, the sections were first deparaffinized and blocked with BSA. Then, the sections were stained with primary antibodies and secondary fluorescent antibodies sequentially. Finally, these sections were observed with CLSM and interesting areas were captured.

### Statistical Analysis

The data are shown as mean ± standard deviation (SD). Statistical differences between two groups were performed using the unpaired Student's *t*‐test and the differences between multiple groups were analyzed using one‐way analysis of variance (ANOVA) (GraphPad Prism5, San Diego, CA, USA). Statistical significance is expressed as *p < 0.05, **p < 0.01, and ***p < 0.001.

## Conflict of Interest

The authors declare no conflict of interest.

## Supporting information

Supporting Information

## Data Availability

Research data are not shared.
