# Supplementary material for: Multifunctional Nanodrug Mediates Synergistic Photodynamic Therapy and MDSCs‐Targeting Immunotherapy of Colon Cancer
Source: Adv Sci (Weinh). 2021 May 21;8(14):2100712. doi: 10.1002/advs.202100712 (PMC8292876; doi:10.1002/advs.202100712)
Supplement: Supplementary file 1 — Supporting Information [file ADVS-8-2100712-s001.pdf]

© 2021 Wiley-VCH GmbH

ADVANCED SCIENCE
Open Access

Supporting Information

for *Adv. Sci.*, DOI: 10.1002/advs.202100712

Multifunctional nanodrug mediates synergistic photodynamic therapy and MDSCs-targeting immunotherapy of colon cancer

Dongbing Ding, Huihai Zhong, Rongpu Liang, Tianyun Lan, Xudong Zhu, Shengxin Huang, Yong Wang, Jun Shao, Xintao Shuai, Bo Wei**

Supporting information

Multifunctional nanodrug mediates synergistic photodynamic therapy and MDSCs-targeting immunotherapy of colon cancer

Dongbing Ding[#]*, Huihai Zhong*[#]*, Rongpu Liang, Tianyun Lan, Xudong Zhu, Shengxin Huang, Yong Wang, Jun Shao, Xintao Shuai*, Bo Wei**

Dr. D. Ding, Dr. R. Liang, Dr. X. Zhu, Dr. S. Huang, Dr. J. Shao, Prof. X. Shuai, Prof. B. Wei

Department of Gastrointestinal Surgery, the Third Affiliated Hospital of Sun Yat-sen University, Guangzhou 510630, China.

Email: shuaixt@mail.sysu.edu.cn; weibo3@mail.sysu. edu. cn.

Dr. H. Zhong, Prof. X. Shuai

PCFM Lab of Ministry of Education, School of Materials Science and Engineering, Sun Yat-sen University, Guangzhou 510275, China.

T. Lan

Central Laboratory, the Third Affiliated Hospital of Sun Yat-sen University, Guangzhou 510630, China.

Dr. Y. Wang

College of Chemistry and Materials Science, Jinan University, Guangzhou 510632, China.

[#]Equal contribution.

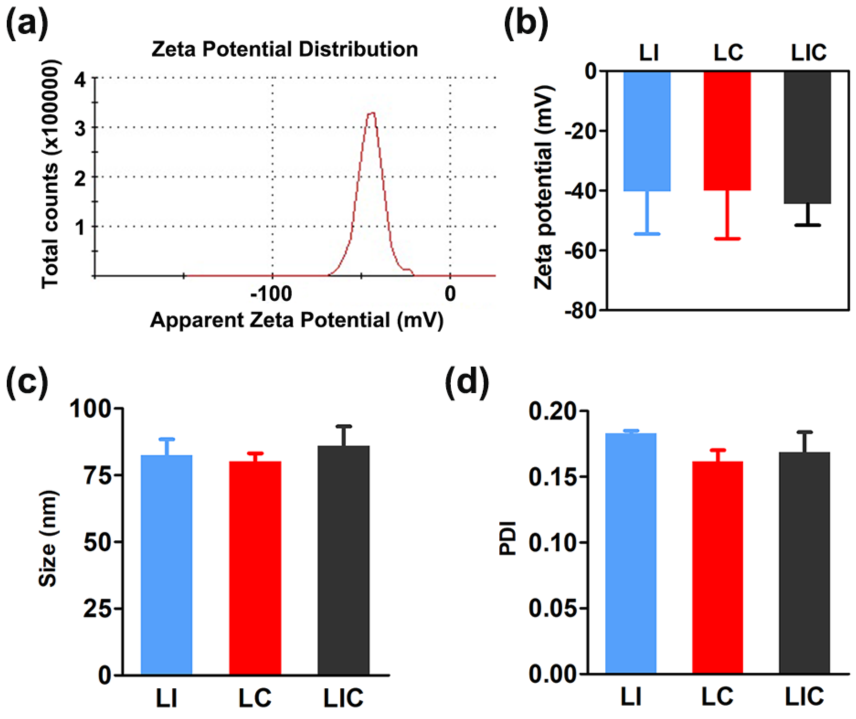

Figure S1. Size, polymer dispersion index (PDI), and Zeta potential of nanodrugs. (a) Zeta potential distribution of LIC. Size (b), PDI (c), and Zeta potential (d) of LI, LC, and LIC (n=3).

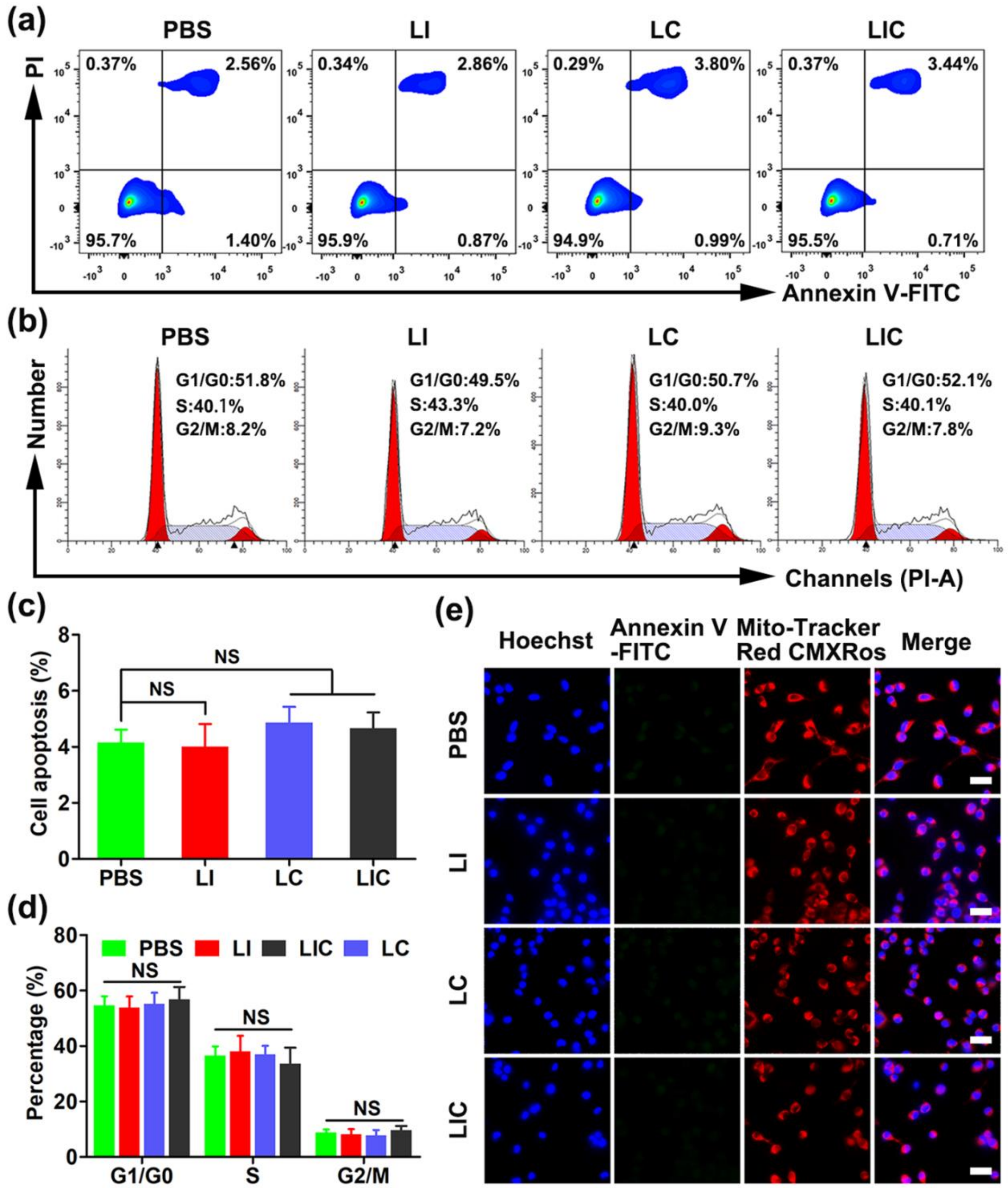

Figure S2. *In vitro* killing effects of nanodrugs on CT26 colon cancer cells without light irradiation. Cell apoptosis (a) and cell cycle distribution (b) after various treatments determined by flow cytometry. Statistical analysis of apoptotic rate (c) and cell cycle distribution (d) of CT26 cells in different groups (n=3). (e) Fluorescence images of Annexin-V (green) staining and staining for change in mitochondrial membrane potential (red) after different treatments by CLSM. Scale bar: 25 μm. NS: no significant difference.

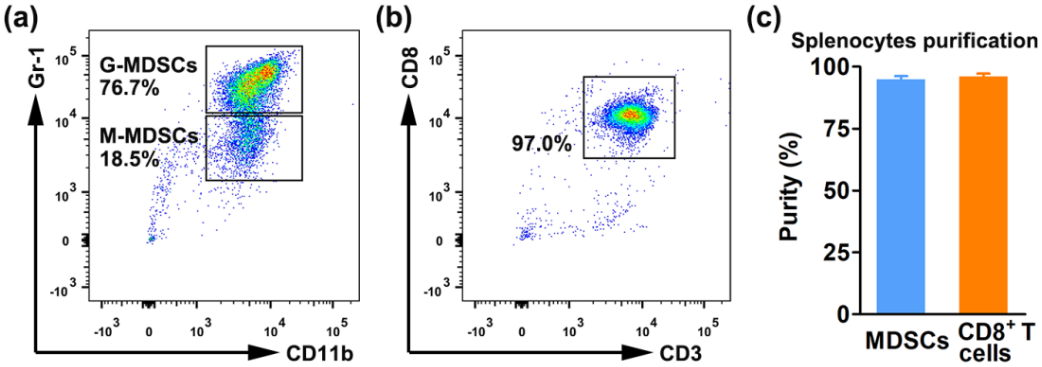

Figure S3. Purification of MDSCs and CD8$^+$ T cells from mouse splenocytes. (a) MDSCs purification from splenocytes of the tumor-bearing mouse using immunomagnetic beads. (b) CD8$^+$ T cells purification from splenocytes of the normal mouse using immunomagnetic beads. (c) Purity of MDSCs and CD8$^+$ T cells after splenocytes purification (n=3).

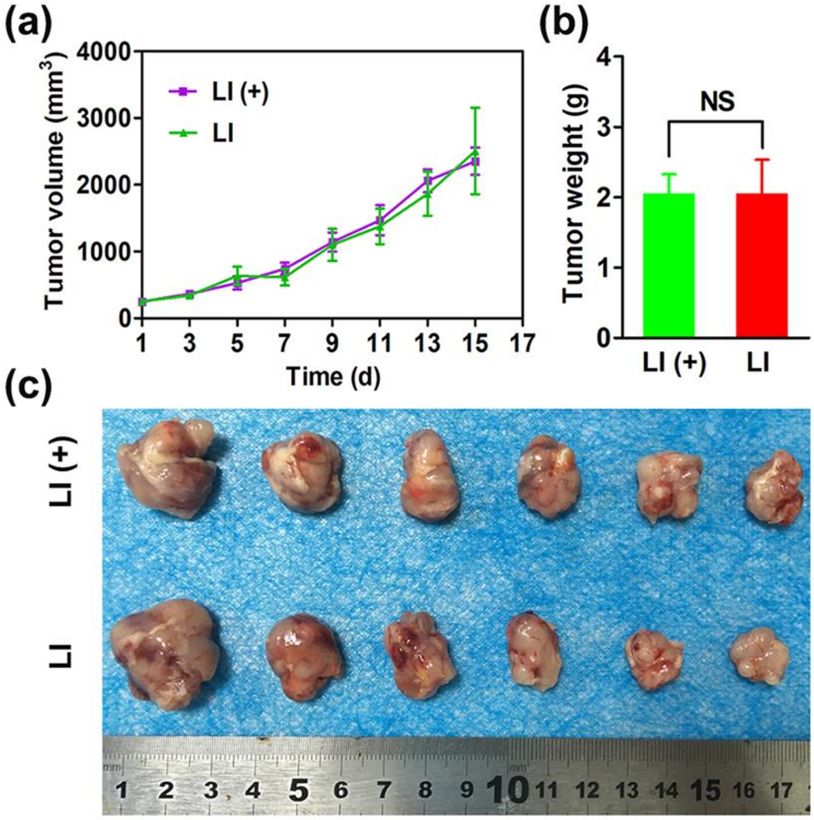

Figure S4. Anti-tumor therapy of LI with or without laser irradiation. (a) Tumor growth curves for mice receiving different treatments. (b) Tumor weights in two therapeutic groups. (c) Photograph of tumors excised from mice receiving LI or LI (+) treatment. Data are shown as mean ± standard error of mean (SEM) (n=6). NS: no significant difference.

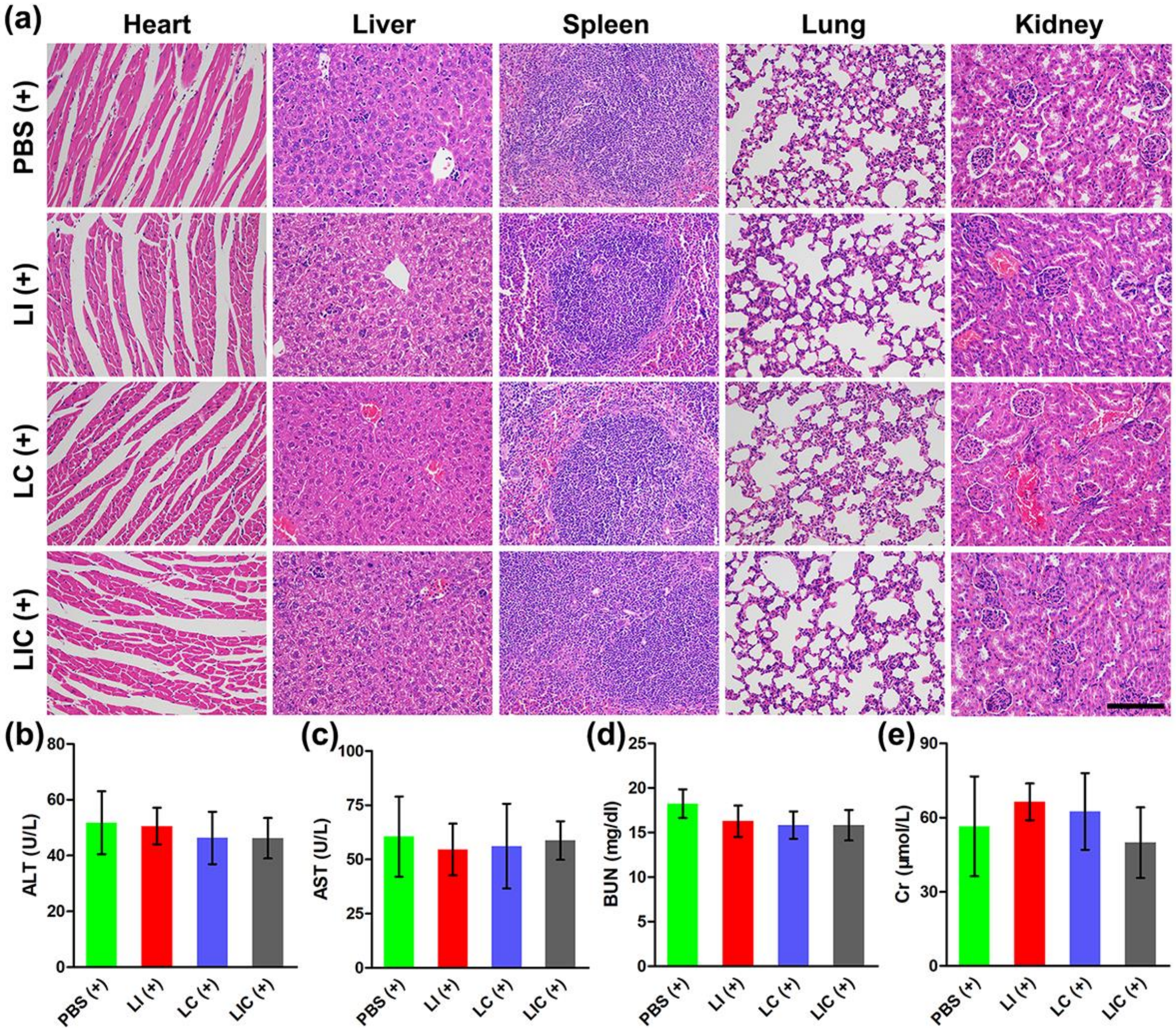

Figure S5. *In vivo* toxicity of nanodrugs to main organs. (a) Pathological changes of heart, liver, spleen, lung, and kidney from tumor-bearing mice receiving various treatments were evaluated by H&E staining. Scale bar: 50 μm. (b-e) Serum levels of liver function (AST, ALT) and kidney function (BUN, Cr) were detected at the end of treatments (n=5).

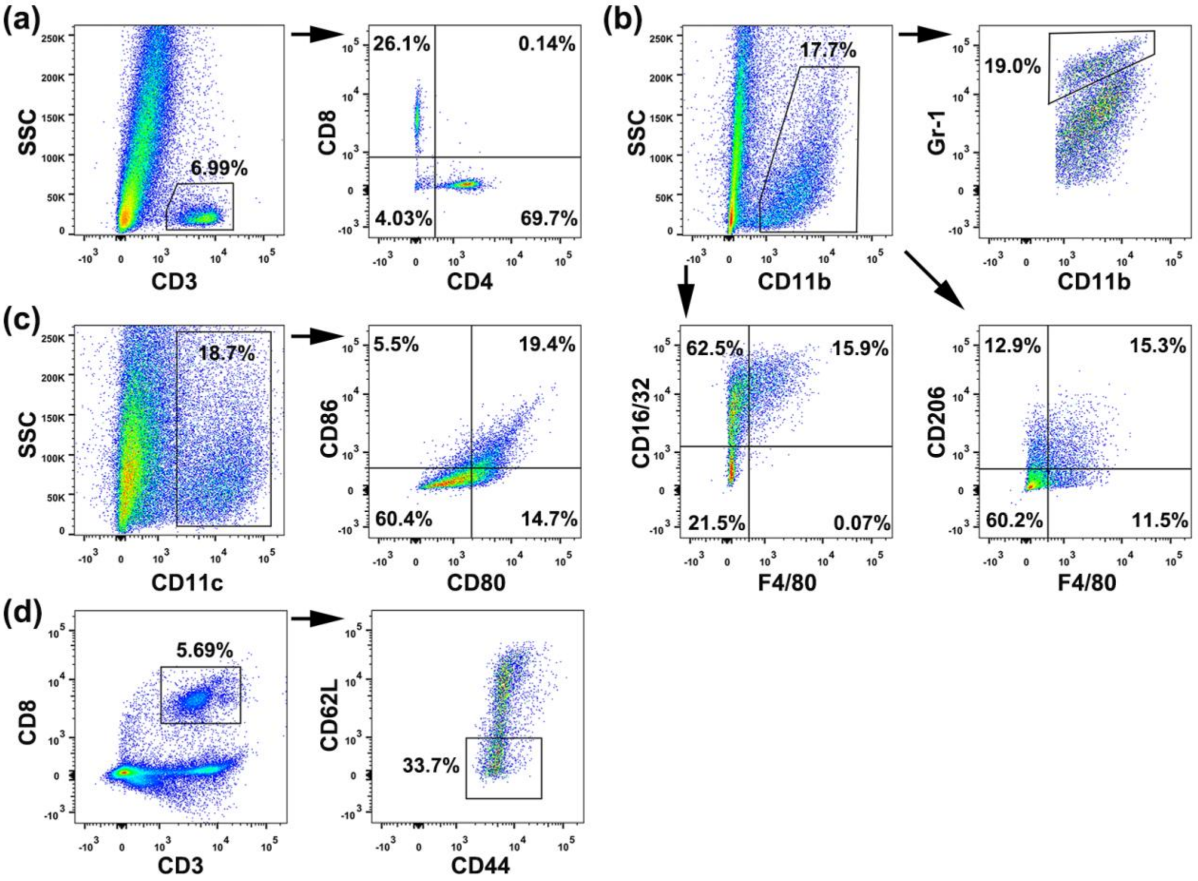

Figure S6. Gating strategies to investigate proportions of various immune cells in tumor or spleen. (a) CD4$^+$ T and CD8$^+$ T cells were gated from CD3$^+$ cells in tumor. (b) MDSCs (CD11b$^+$Gr-1$^+$), M1-like tumor-associated macrophages (CD11b$^+$F4/80$^+$CD16/32$^+$) and M2-like tumor-associated macrophages (CD11b$^+$F4/80$^+$CD206$^+$) were gated from CD11b$^+$ populations in tumor. (c) Mature DCs (CD80$^+$CD86$^+$) were gated on CD11c$^+$ subsets in tumor. (d) Effector memory T cells (CD3$^+$CD8$^+$CD44$^+$CD62L$^-$) were gated from CD3$^+$CD8$^+$ T cells in spleen.

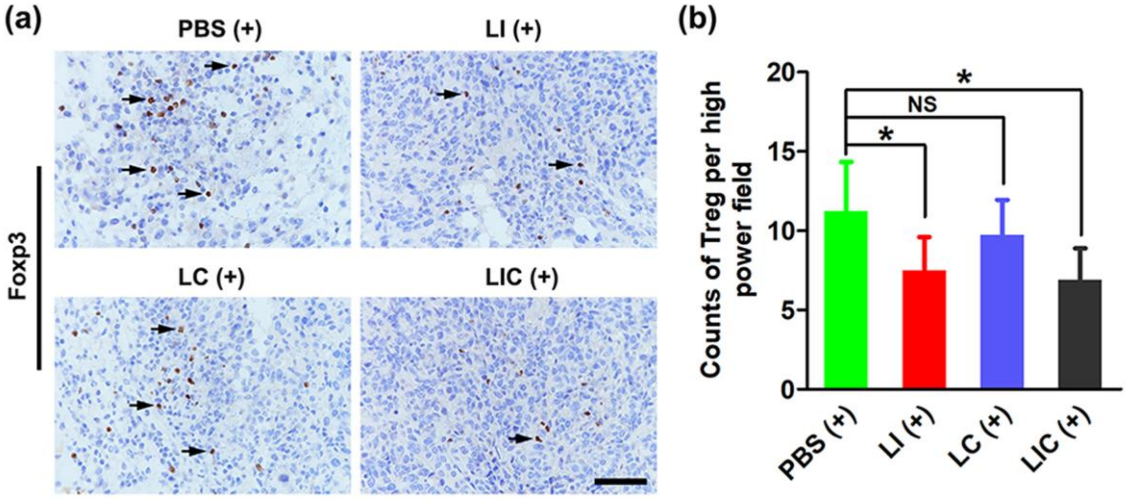

Figure S7. Nanodrugs decreased the number of Tregs in CT26 tumors. (a) Immunohistochemical staining of Tregs ($Foxp3^+$) in CT26 tumors after different treatments. Scale bar: 50 µm. (b) Corresponding quantification analysis of Foxp3 expression in CT26 tumors in different treatment groups. *$p < 0.05$, NS: no significant difference.

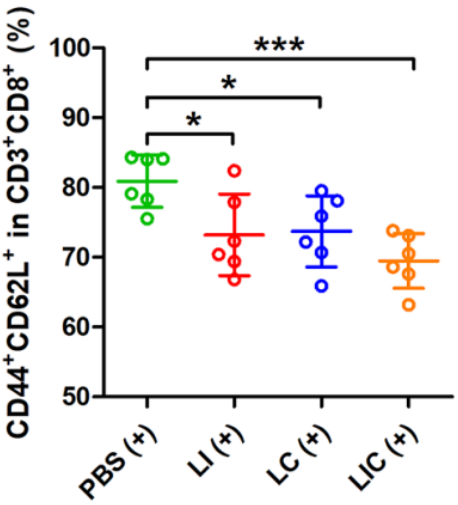

Figure S8. Nanodrugs decreased the percentages of central memory T cell (T_{CM}) in spleen. Flow cytometry analysis of T_{CM} ($CD3^+CD8^+CD44^+CD62L^+$) in spleen after various treatments (n=6). *$p < 0.05$, ***$p < 0.001$.

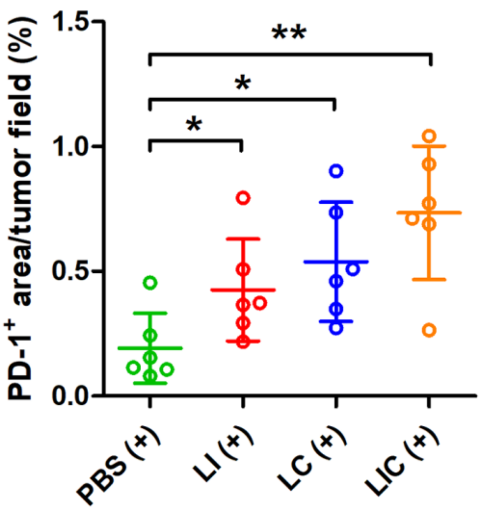

Figure S9. Nanodrugs increased PD-1 expression in CT26 tumors. Quantitative analysis of PD-1 immunofluorescence-positive area in tumors after various treatments (n=6). *p < 0.05, **p < 0.01.

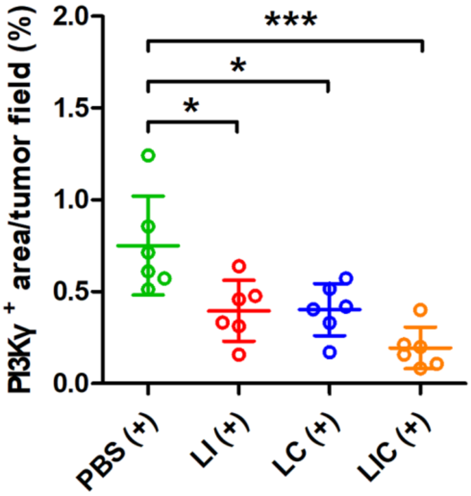

Figure S10. Nanodrugs decreased PI3Kγ expression in CT26 tumors. Quantitative analysis of PI3Kγ immunofluorescence-positive area in tumors after various treatments (n=6). *p < 0.05, ***p < 0.001.

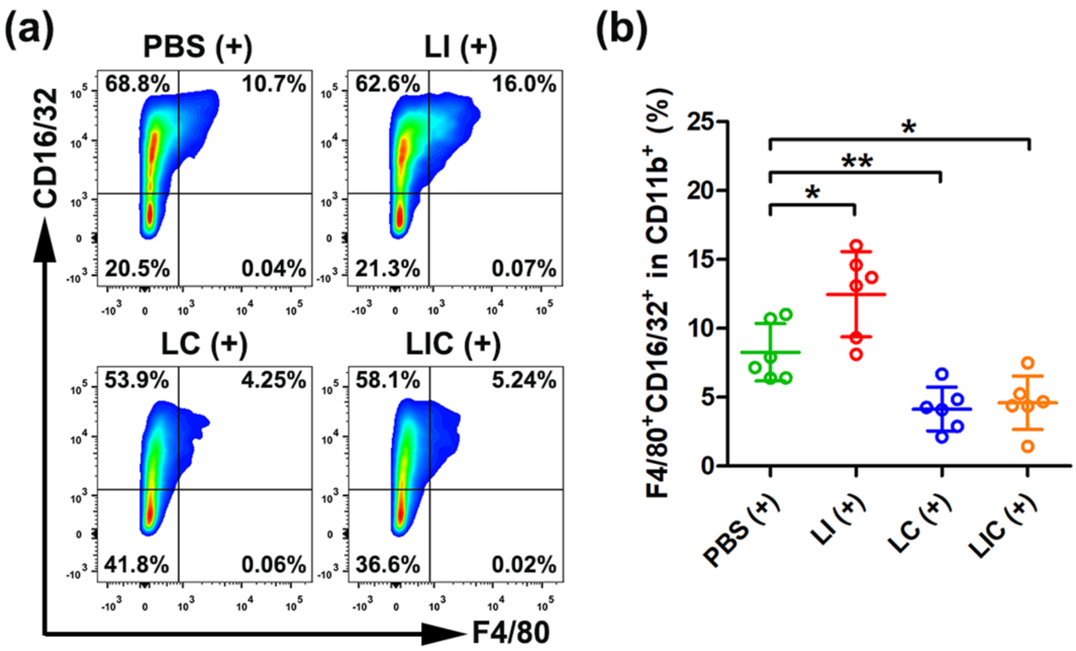

Figure S11. The effect of nanodrugs on M1-like tumor-associated macrophages (TAMs) in CT26 tumors. (a) M1-like TAMs in tumor tissues after different treatments determined by flow cytometry analysis (gated on $CD11b^+$ cells). (b) Statistical analysis of M1-like TAMs ($CD11b^+F4/80^+CD16/32^+$) ratio in $CD11b^+$ cells (n=6). *$p < 0.05$, **$p < 0.01$.

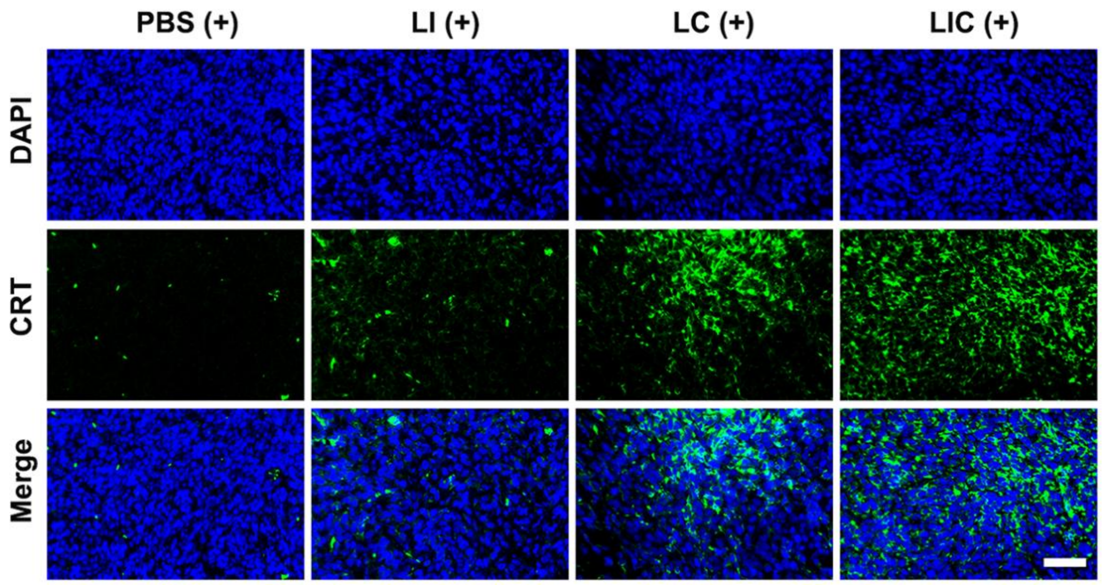

Figure S12. CRT expression in CT26 tumors from mice receiving different treatments. Immunofluorescent staining for the nucleus (blue) and CRT (green) in CT26 tumor sections after different treatments. Scale bar: 50 μm.

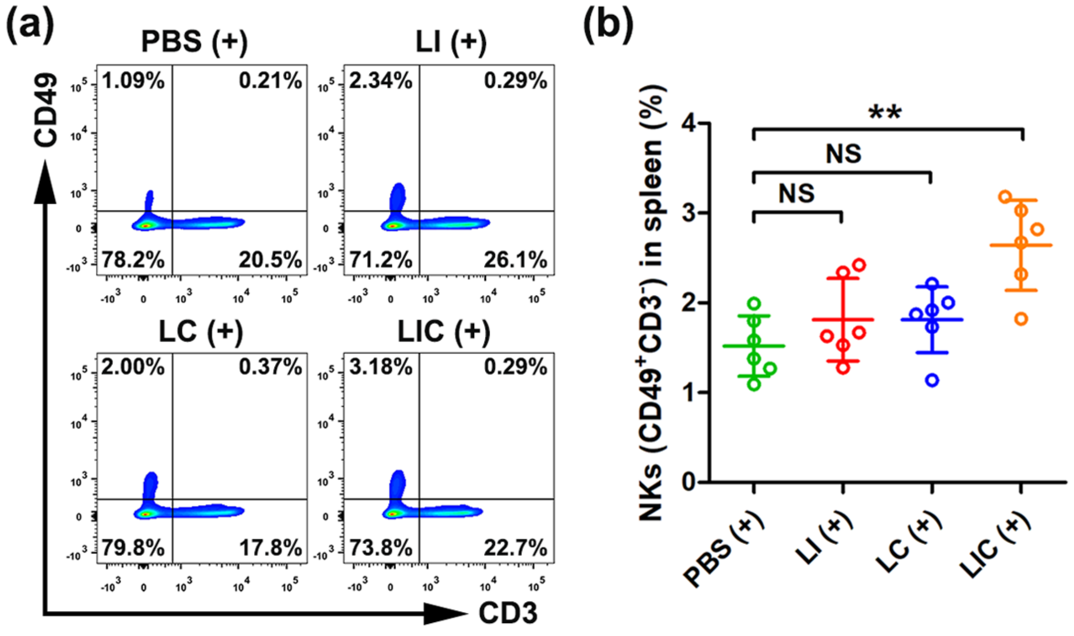

Figure S13. Nanodrugs promoted the activation of NK cells (NKs) in spleen. (a) NKs in spleen after different treatments determined by flow cytometry (gated on $CD3^{-}CD49^{+}$ cells). (b) Quantification analysis of the percentages of NKs in spleen after different treatments (n=6). ***p* < 0.01, NS: no significant difference.
